# Clinical efficacy of exercise therapy for lumbar disc herniation: a systematic review and meta-analysis of randomized controlled trials

**DOI:** 10.3389/fmed.2025.1531637

**Published:** 2025-03-28

**Authors:** Shaojie Du, Zeyu Cui, Shurui Peng, Jieyu Wu, Jinhai Xu, Wen Mo, Jie Ye

**Affiliations:** ^1^Shanghai University of Traditional Chinese Medicine, Shanghai, China; ^2^Department of Orthopedics and Traumatology, Longhua Hospital Shanghai University of Traditional Chinese Medicine, Shanghai, China; ^3^Shanghai Geriatric Institute of Chinese Medicine, Shanghai University of Traditional Chinese Medicine, Shanghai, China

**Keywords:** lumbar disc herniation, exercise therapy, clinical trial, meta-analysis, traditional Chinese exercise

## Abstract

**Background:**

Lumbar disc herniation (LDH) is mainly characterized by pain and neurological dysfunction resulting from rupture of the fibrous ring of the lumbar disc. The incidence of LDH is increasing year by year, mainly owing to long-term sitting and immobility, improper posture, and reduced physical activity during study and work. The incidence of LDH in young people aged 18–35 years has shown a significant upward trend. LDH is one of the most common causes of back pain, affecting approximately 1–3% of the global population, and it is mainly concentrated in people aged 30–50 years. Exercise therapy, as an economically effective treatment method, can enhance the strength of core muscle groups and effectively alleviate the symptoms of LDH. However, strong evidence that proves the effectiveness of exercise therapy for LDH is still lacking. We conducted a meta-analysis of relevant clinical studies and used statistical methods to scientifically explore the effectiveness of exercise therapy for LDH.

**Methods:**

Four databases were searched, including PubMed, Embase, Web of Science, and Ovid. The search period ended on August 24, 2024. Comparisons were made between the group that used exercise therapy (treatment group) and the group that did not (control group). If heterogeneity among the studies was strong, sensitivity analysis was performed.

**Results:**

Of the 580 articles initially identified, eight (encompassing 611 patients with LDH) were included in the analysis. The results of the meta-analysis demonstrated that the treatment group exhibited superior outcomes in terms of Visual Analog Scale score, Oswestry Disability Index, range of motion, McSensory score, McTotal score, and Short Form-36 score compared with the control group.

**Conclusion:**

Exercise therapy is an economical, effective, and simple adjuvant therapy for patients with LDH. Exercise therapy enhances core muscle strength and lumbar stability, relieves lower back and leg pain, and improves patients’ quality of life. However, no consensus on the use of exercise therapy for LDH currently exists, and there are no specific recommendations for exercise intensity, frequency, and activity that are suitable for patients with LDH. More high-quality clinical studies are needed to validate the application of exercise therapy as a treatment for LDH.

**Systematic Review Registration:**

The protocol was registered on PROSPERO (CRD42024586775).

## 1 Introduction

Lumbar disc herniation (LDH) is a condition in which the fibrous annulus of the lumbar disc ruptures, causing the nucleus pulposus to protrude and compress the spinal nerves and cauda equina, resulting in a range of clinical symptoms, including pain and neurological dysfunction (1). LDH results from degenerative changes in the annulus fibrosus (2). These changes may include a decrease in the water concentration in the medulla, an increase in the percentage of type I collagen in the medulla and the inner fibrous ring, damage to collagen and extracellular material (3), and upregulation of matrix metalloproteinase expression. With the change of tissue composition, cell cycle and inflammation stimulation, apoptosis and the activity of inflammation-related signaling pathways may be induced which can culminate in the clinical manifestations of LDH, including lower back pain, lower-extremity numbness, and lower-extremity distraction.

Diagnostic methods for LDH mainly include X-ray, myelography, computed tomography, and magnetic resonance imaging (MRI). Currently, MRI is the most effective method for the diagnosis of intervertebral disc herniation (1). MRI has revealed that the most common sites of LDH are the lumbar spine regions L4–L5 and L5–S1 (4).

The clinical treatment strategy is determined on the basis of the site of LDH occurrence. Current treatment strategies for LDH are categorized into surgical intervention and conservative treatment. Conservative treatment is the first choice for most patients with their first diagnosis of LDH. The routine course of treatment lasts at least 6 weeks and mainly involves bed rest, medication, exercise therapy, epidural injection, lumbar traction, and traditional Chinese medicine (4, 5). The efficacy assessment of conservative treatment is based on the patient’s self-perception and resorption of the herniated material. Spontaneous resorption with conservative treatment for LDH was first reported in 1984, confirming the feasibility of conservative treatment for LDH (6). In recent years, LDH resorption has received increasing attention from researchers. A retrospective analysis showed that 320 of 409 patients with LDH were treated conservatively, and herniated resorption was observed in 189 patients (59.06%) (7). Although the composition of the intervertebral disc may affect the likelihood of resorption owing to differences in the annulus fibrosus, cartilaginous endplates, and nucleus pulposus, there is still evidence supporting the efficacy of conservative treatment for LDH (8).

Exercise therapy is a form of conservative treatment that has been shown to be effective for LDH. As Huber’s team reported, muscle strengthening and training reduced LDH symptoms significantly compared to controls, especially by targeting core muscle groups and improving coordinated physical stability. Therefore, core strengthening training is an indispensable part of LDH rehabilitation (9). Exercise therapy increases muscle strength and endurance, especially by targeting core muscles, such as the transversus abdominis and multifidus, to improve coordination and trunk stability. Core-strengthening exercises are an integral part of rehabilitation for LDH. Strengthening the core muscles is an important factor in the regulation of intraabdominal pressure during spinal motion, which contributes to spinal stability and reduces the load on the lumbar spine (10), as well as enhancing the stability and mobility of the sacroiliac joints by controlling pelvic motion (11). Traditional Chinese exercises, which combine body adjustment and breathing rhythm, are an important part of traditional Chinese medicine culture. Since ancient times, the traditional Chinese movement combines the “body” and the “mind,” regulates the meridians, connects the organs, and makes the internal environment of the body reach a balanced state (12). Therefore, traditional Chinese exercise is included as part of exercise therapy. Its application in spinal disorders is widespread. For instance, Yao Fei’s team is dedicated to the study of tuina and traditional Chinese exercise, using Tuina combined with Yijinjing to treat patients with neck pain (13). Tuina is a kind of external treatment of traditional Chinese medicine, which is different from traditional Chinese hurbs. It is used to treat related diseases combined with the theory of meridians, by using the hand, elbow and other parts of the human body surface, as a physical therapy characterized by mechanics. Nowadays, massage science attaches great importance to the theory and application of modern biomechanics. Yijinjing, a traditional Chinese exercise, emphasizes the coordination of body, mind and breath. This is a simple, easy and universal physical and mental exercise. Their team found that this approach was more effective than Tuina alone in terms of pain, functional recovery, and anxiety. Zhou et al. (14) clarified the method and indications for spinal fine-tuning manipulation for the treatment of LDH. Spinal fine-tuning manipulation is a kind of tuina technique, which has evolved through the development of modern Chinese medicine masters. It is a feasible and effective chiropractic procedure to the patient’s informed permission, in patients with LDH, excluding those who have contraindications such as osteoporosis, sever cases, LDH postoperative patients or lumbar spinal stenosis. They screened four traditional movements, namely the cloud-handed spinal retraction method, Bawang Ju Ding, Lou Xi Ao Bu, and Dao Nian Hou, to form the basic technical specification of spinal fine-tuning manipulation combined with traditional Gongfu for the treatment of LDH. Moreover, Liu et al. (15) studied the application of the strong tendon Gong method in patients with LDH, revealing that under the supine leg raising strong tendon force Gong method, the thickness of the multifidus muscle belly increased with an increase in the degree of supine leg raising. Under the two-handed foot-climbing and kidney–waist-fixing Gong method, the multifidus muscle belly became thinner with an increase in the two-handed foot-climbing and forward-flexion angles. These findings indicated that the strong tendon Gong method can exercise the multifidus muscle by changing its stretch state, in turn improving the symptoms of LDH.

This meta-analysis evaluates the clinical efficacy of exercise therapy for LDH. This analysis examined the adjunctive role of exercise therapy in non-surgical management strategies, demonstrating its viability and safety profile under comprehensive multimodal care models. These exercises are suitable for patients with mild LDH who can be performed aerobic exercise. We only discussed aerobic exercise and did not explore the effect of other exercise types on LDH. Strenuous or difficult exercise has been ruled out. The results will be used as a basis for relevant prospective predictions and research advances to understand the mechanisms underpinning the effects of exercise therapy in LDH.

## 2 Materials and methods

### 2.1 Search strategy

We searched four databases (PubMed, Embase, Web of Science, and Ovid) to identify randomized controlled trials published from the database inception to August 24, 2024. The search strategy is shown in Table 1. The protocols used in this systematic evaluation are registered at PROSPERO (CRD42024586775). The included studies were restricted to those published in the English language.

**Table 1:** Literature search strategy.

Database	Number	Search strategy
PubMed	-	((yoga) OR (exercise) OR (Yijinjing) OR (shaolin internal qigon)) AND ((lumbar disc herniation) OR (LDH)) AND (clinical trial)
Web of Science	#1	(TS = (yoga))ORTS = (exercise)ORTS = (Yijinjing)ORTS = (shaolininternalqigon)
	#2	TS = (lumbar disc herniation)ORTS = (LDH)
	#3	TS = (clinical trial)
	#4	#3AND#2AND#1
Ovid	1	(yoga or exercise or Yijinjing or shaolin internal qigon).af.
	2	(lumbar disc herniation or LDH).af.
	3	clinical trial.af.
	4	1and2and3
Embase	#1	'clinical trial'exp OR 'clinical trial' OR (('clinical'/exp OR clinical) AND ('trial'/exp OR trial))'
	#2	'lumbar disc herniation'/exp OR 'lumbar disc herniation' OR 'ldh'/exp OR 'ldh'
	#3	'yoga'/'exp OR 'yoga' OR 'exercise'/exp OR 'exercise' OR 'yilinjing'/exp OR 'yijinjing' OR 'shaolin internal qigon'
	#4	#1AND#2AND#3

### 2.2 Inclusion and exclusion criteria

All randomized controlled trials with complete information on interventions and outcomes, published in the English language, and for which the primary disease of study was LDH were included. Because exercise therapy is an adjunct therapy, articles that studied exercise combined with other therapies, such as Tuina, to explore the effectiveness of exercise therapy were included. Moreover, the included patients were required not to have changed their medication regimen 1 month before or during the intervention. No limitations were set for the comparator. Specific scales, including the Visual Analog Scale (VAS), Oswestry Disability Index (ODI), range of motion (ROM), McSensery, McTotal, and Short Form (SF)-36 scales, were used after treatment to assess the effectiveness of exercise therapy for LDH.

**Figure 1:**
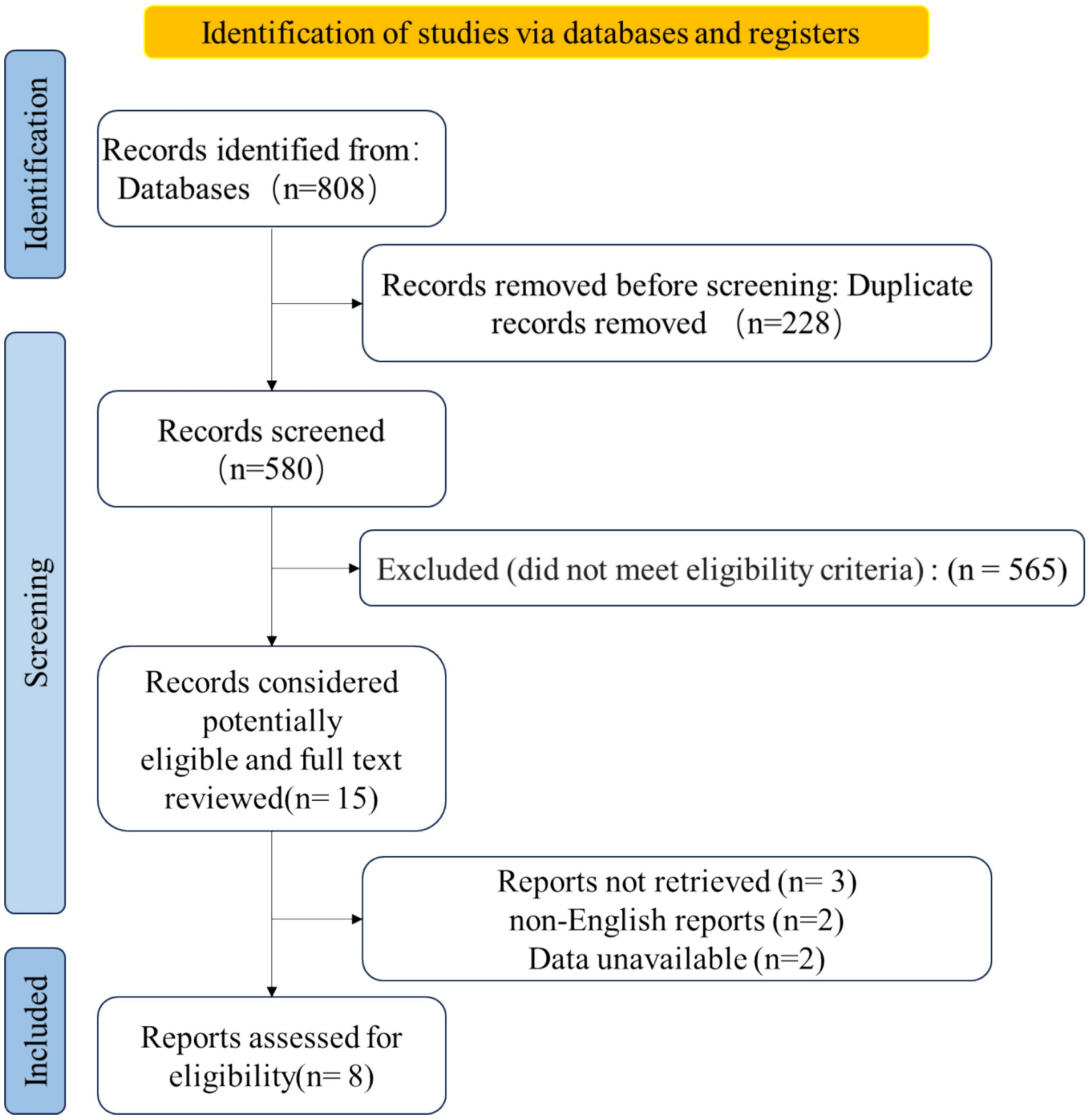
Flowchart detailing the process of study selection.

Figure 1 illustrates the process of literature selection. First, the retrieved literature was imported into EndNoteX9, and the automatic review function was used to eliminate duplicate articles. Second, three independent investigators (DSJ, WJY, and PSR) manually screened for unrecognized duplicates, including duplicates from different publications and multilingual publications, as well as reports on different aspects of the same study. These three investigators then screened the titles and abstracts of the articles to select eligible studies based on the study type, intervention/comparator, and outcomes. Third, full-text assessment was performed by two investigators (DSJ and WJY), and articles were excluded based on the eligibility criteria. The eligibility criteria were as follows: (1) the disease under study was LDH; (2) the study type was a clinical randomized controlled trial; (3) the study used exercise therapy in the treatment group but not in the control group over a period of time starting from the beginning of the clinical trial and clinical assessment at the relevant time point; and (4) studies in which no surgery was used to treat LDH. Any disagreements in literature selection were resolved by consensus.

### 2.3 Risk-of-bias assessment

Two researchers (DSJ and PSR) independently assessed the methodological quality of the included randomized controlled trials according to the Cochrane risk-of-bias tool, and each article was categorized as low, high, or unclear risk. The seven items evaluated for bias in each trial included (1) randomization sequence generation, (2) allocation concealment, (3) blinding of subjects and personnel, (4) blinding of outcome assessment, (5) incomplete outcome data, (6) selective reporting, and (7) other biases.

The included trials were classified as low, high, or moderate quality. Trials were considered to be of low quality if randomization or allocation concealment had a high risk of bias, regardless of the risk of the other items. Trials were considered to be of high quality when randomization and allocation concealment had a low risk of bias and all other items were assessed to have a low or unclear risk of bias. Trials that did not meet the high or low risk criteria were considered to be of moderate quality.

### 2.4 Data extraction

The reviewers (DS and PS) rigorously extracted the following information from the included studies: authors, year of publication, trial design, country, type of intervention, intervention characteristics, number of participants, age of participants, and outcomes. For continuous variables, if the article had multiple subgroups of outcomes under the same outcome indicator (e.g., VAS scores including lumbar VAS scores and lower-extremity VAS scores), they were treated according to the subgroup combination formula. If the sample size of subgroup A was N1, the mean was M1, and the standard deviation (SD) was SD1, and the sample size of subgroup B was N2, the mean was M2, and the SD was SD2, with a combined sample size (N = N_1_ + N_2_) and mean [M = (N_1_M_1_ + N_2_M_2_) ÷ (N_1_ + N_2_)], the formula to calculate the SD was as follows:.


$$SD=\sqrt{\frac{\left(N_{1}-1\right){SD}_{1}^{2}+\left(N_{2}-1\right){SD}_{2}^{2}+\frac{N_{1}N_{2}}{N_{1}+N_{2}}\left(M_{1}^{2}+M_{2}^{2}-2M_{1}M_{2}\right)}{N_{1}+N_{2}-1}}$$

If multiple subgroups of data needed to be merged, data from two subgroups were merged according to the above formula, and the resulting data were merged with the third subgroup. Any disagreements among the reviewers were resolved by consensus or by consulting a third reviewer (WJY).

### 2.5 Data analysis and statistical methods

Data analysis was performed using Review Manager 5.4. Continuous data are expressed as the mean difference with 95% confidence interval. Heterogeneity was assessed using the I^2^ test. According to the recommendations of the Cochrane Handbook 5.1.0 (16), I^2^ was categorized as follows: <25% indicated mild heterogeneity; 25–50% indicated moderate heterogeneity; and >50% indicated strong heterogeneity. Owing to the strong heterogeneity among exercise therapy interventions, the random-effects model was used. For studies with strong heterogeneity, sensitivity analysis was performed using Stata 14 software to assess the stability of the results.

## 3 Results

### 3.1 Study selection

As shown in Figure 1, the database search identified 808 unique records. Of these, 228 studies were duplicates, which were excluded. Next, 565 studies were excluded based on the screening criteria. Therefore, 15 relevant studies were retrieved. Of these, three articles could not be retrieved, two were not written in English, and two had unavailable data. These studies were excluded, leaving eight studies to be included in the analysis (17–24).

### 3.2 Characteristics of the included studies

Table 2 shows the characteristics of the eight studies (17–24), which were published between 2005 and 2024 and involved 611 participants. All participants were between the ages of 18 and 65. All of the trials were published in the English language. Three studies explored traditional exercise, three evaluated spinal exercise, and two evaluated instrument-assisted exercise. All trials reported overall symptom improvement. No trials reported data on cost-effectiveness, number of recurrent episodes, or adverse events.

**Table 2:** Characteristics of the included studies.

Number	Records	Title	Treatment	Sample size		Baseline						Follow-up time	Intervention program		Outcome measure
				Treatment group	Control group	Treatment group			Control group						
						Age	Male	Female	Age	Male	Female		Treatment group	Control group	Outcome measures
1	Xin et al. (14)	Effect of traditional Chinese exercise combined with massage on pain and disability in patients with lumbar disc herniation: A multi-center, randomized, controlled, assessor-blinded clinical trial	Tai Chi	130	129	44.36	64	66	51.77	58	71	6 weeks	Tai Chi+TCM therapy for 6 weeks	TCM therapy for 6 weeks	ODI; VAS; ROM; SF-MPQ;
2	Yildirim et al. (21)	The effect of instrument-assisted soft tissue mobilization in lumbar disc herniation: A randomized controlled trial	instrument-assisted soft tissue mobilization	30	30	46.33	15	15	57.03	10	20	——	instrument-assisted soft tissue mobilization and conventional physiotherapy for 4 weeks	conventional physiotherapy for 4 weeks	VAS; ROM; ODI; SF-36
3	Yildirim et al. (22)	The Effect of a Stretch and Strength-Based Yoga Exercise Program on Patients with Neuropathic Pain due to Lumbar Disc Herniation	Yoga Exercise	24	24	37.75	0	24	38.21	0	24	6 months	Yoga Exercise for 12 weeks	none	VAS; ODI; passive knee extension; Schober; McLikert; McSensory; McPerception; McTotal; DN4; LANSS
4	Danazumi et al. (18)	Effects of spinal manipulation or mobilization as an adjunct to neurodynamic mobilization for lumbar disc herniation with radiculopathy: a randomized clinical trial	Mulligan’s spinal mobilization with leg movement	20	20	38.98	13	7	41.01	15	5	52 weeks	Mulligan’s spinal mobilization with leg movement for 12 weeks	spinal manipulative therapy for 12 weeks	VAS; RMDQ; SBI;SFI; TUG; GROC
5	Khaniand Jahanbin (17)	A Randomized Controlled Trial on the Effect of Repeated Lumbar Traction By A Door-mounted Pull-up Bar on the Size and Symptoms of Herniated Lumbar Disk	repeated lumbar traction by suspension from a pull-up bar	25	25	35.3	13	12	36.7	13	12	——	repeated lumbar traction by suspension from a pull-up bar for 2 months	medication	VAS;MRI
6	Taşpınar et al. (20)	The effects of Pilates on pain, functionality, quality of life, flexibility and endurance in lumbar disc herniation	Pilates	27	27	50.3	14	13	50.1	10	17	——	Pilates for 6 weeks	none	ODI; SF-36; VAS
7	França et al. (19)	Motor Control Training Compared With Transcutaneous Electrical Nerve Stimulation in Patients With Disc Herniation With Associated Radiculopathy	motor control training	20	20	43.1	8	12	46.8	7	13	——	motor control training for 8 weeks	transcutaneous electrical nerve stimulation for 8 weeks	VAS; ODI; McGill Pain Questionnaire
8	Bakhtiary et al. (24)	Lumbar stabilizing exercises improve activities of daily living in patients with lumbar disc herniation	Lumbar stabilizing exercises (LSE)	30	30	33	——	——	32.6	——	——	——	a 4 week LSE	none	VAS; Trunk flexion; SLR angle; activities of daily living

Follow-up refers to the follow-up duration after the end of the treatment. VAS, Visual Analog Scale; ODI, Oswestry Disability Index; ROM, range of motion; SF-MPQ, Short Form-McGill Pain Questionnaire; SF-36, Short Form-36; RMDQ, Roland-Morris Disability Questionnaire; DN4, Douleur Neuropathique 4 Questions; LANSS, Leeds Assessment of Neuropathic Symptoms and Signs; SBI, Sciatica Bothersomeness Index; SFI, Sciatica Frequency Index; TUG, Timed Up and Go; GROC, Global Rating of Change.

### 3.3 Risk-of-bias assessment

**Figure 2:**
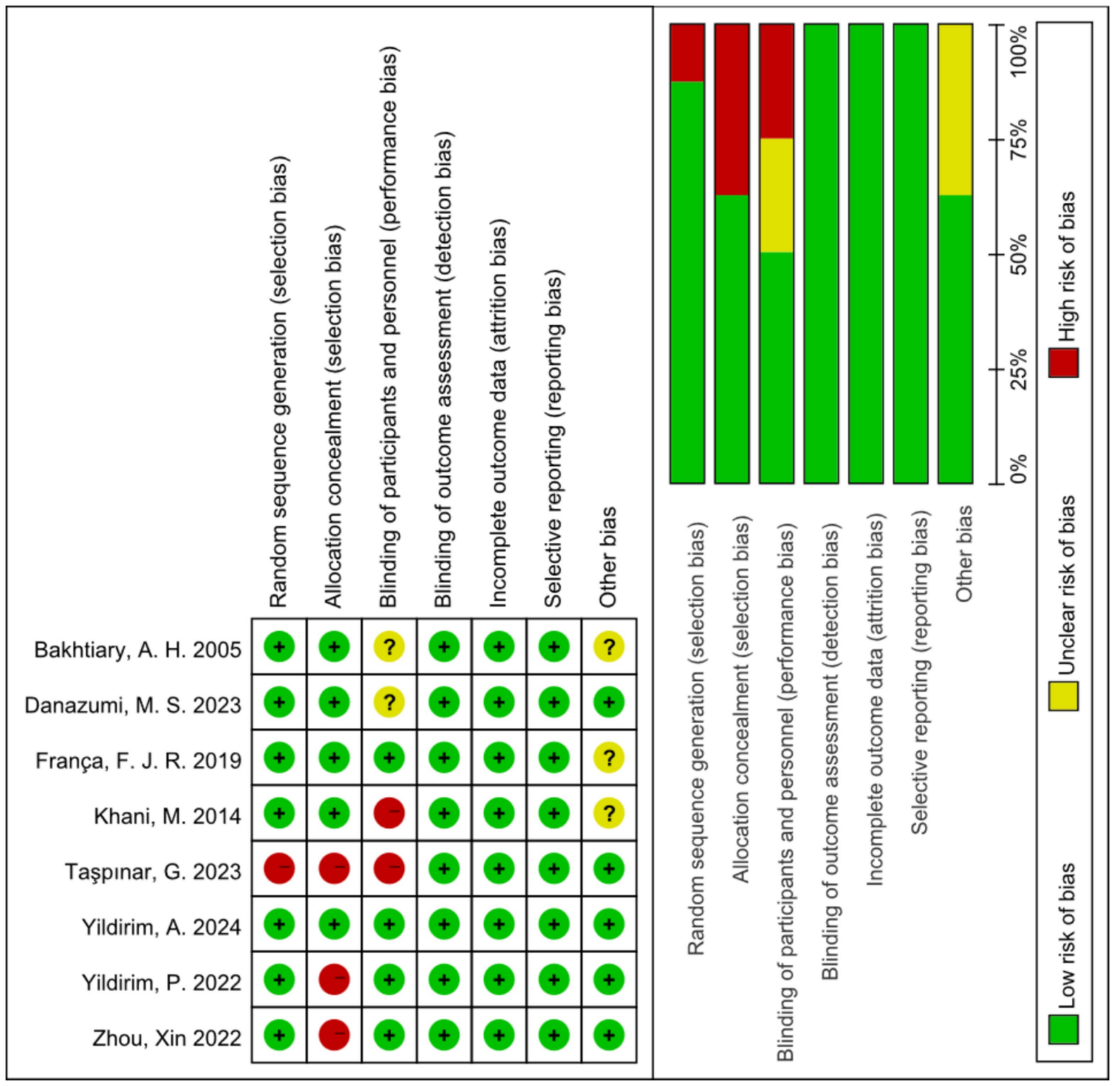
Risk of bias summary.

With regard to random sequence generation, seven trials used random number tables or statistical software. These studies were considered to have a low risk of bias. One trial had a high risk of bias because it did not use randomized sequence generation. Three trials had a high risk of bias for allocation concealment because they did not adhere to random allocation principles. Two trials had a high risk of bias due to difficulties in ensuring the blinding of participants and staff to two different intervention groups. Two trials did not specify blinding of participants and staff and were therefore assessed as having an unclear risk of bias. Eight trials reported blinding of the outcome assessments and were therefore assessed as having a low risk of bias. Eight trials had no missing participants and were therefore assessed as having a low risk of bias. For selective reporting, eight trials reported outcomes according to the methodology outlined in the original study and were therefore assessed as having a low risk of bias. Regarding other potential sources of bias, five trials that disclosed the source of funding were considered to have a low risk of bias. Three trials that did not report the funding source were assessed as having an unclear risk of bias (Figure 2).

### 3.4 Qualitative meta-analysis

#### 3.4.1 VAS score

**Figure 3:**
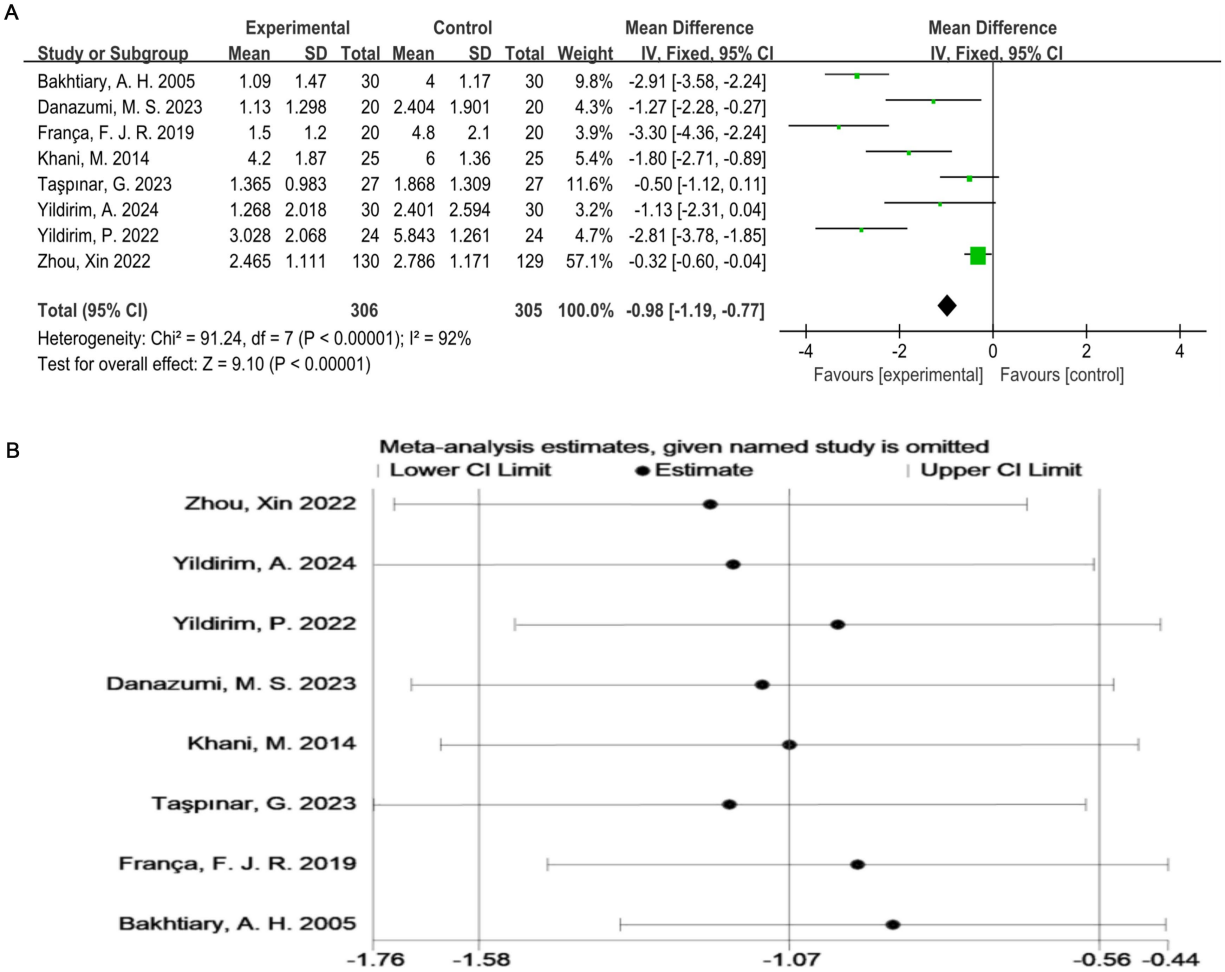
**(A)** Direct pairwise random-effects meta-analysis of the VAS score. **(B)** Sensitivity analysis of the VAS score. VAS, Visual Analog Scale.

As shown in Figure 3A, all studies (17–24) used the VAS score as an outcome indicator. Overall, 611 subjects participated, and the exercise therapy group showed a significant improvement in the pain score compared with the control group. Exercise therapy significantly relieved pain in patients with LDH (*p* < 0.00001). The meta-analysis applying the random-effects model revealed that the VAS score of the treatment group was lower than that of the control group. However, there was a high degree of heterogeneity among the studies (I^2^ = 92%, *p* < 0.0001). To validate the results, a sensitivity analysis was performed, which showed that the results were robust despite the considerable heterogeneity among the studies (Figure 3B).

#### 3.4.2 ODI

**Figure 4:**
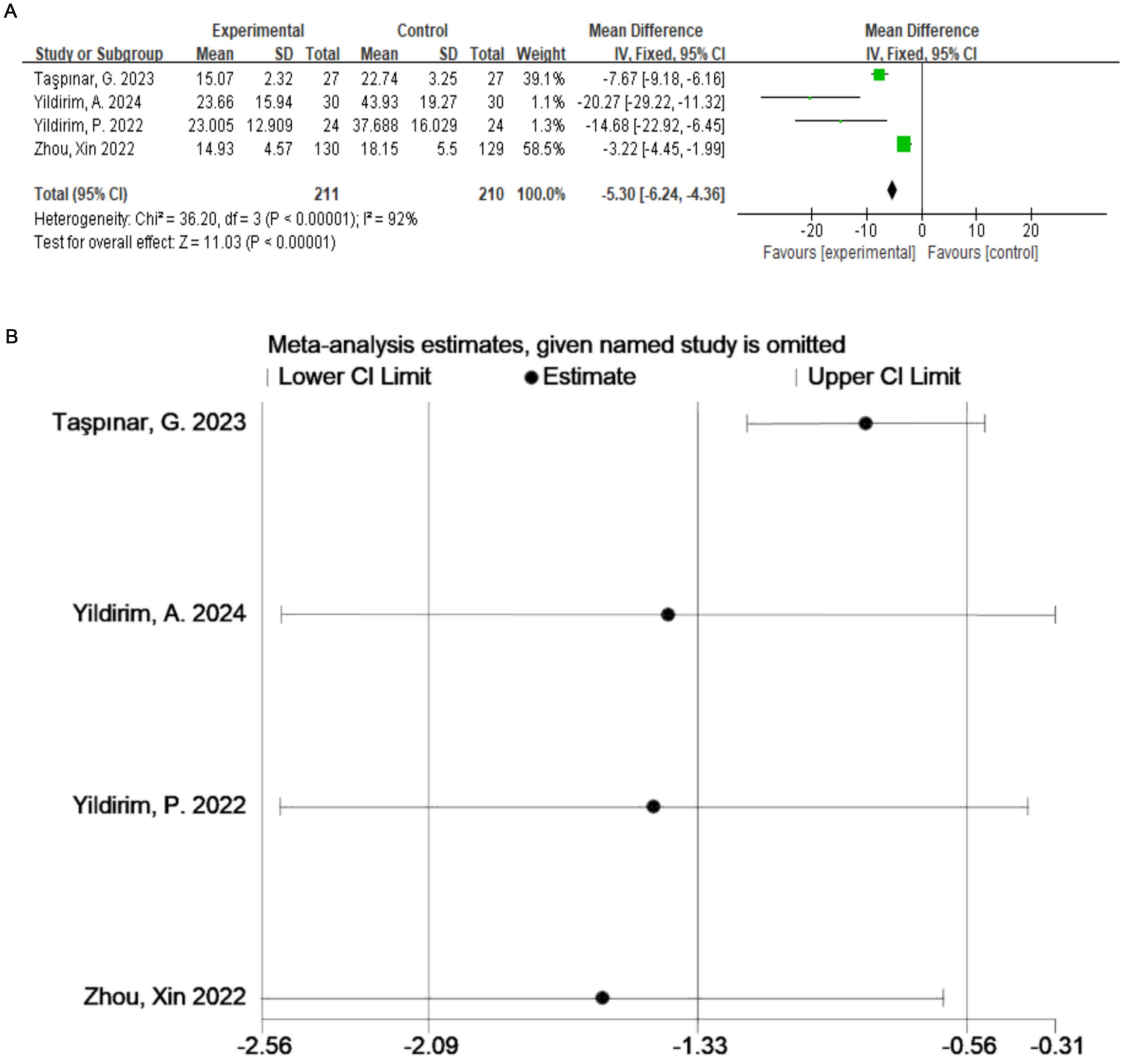
**(A)** Direct pairwise random-effects meta-analysis of the ODI. **(B)** Sensitivity analysis of the ODI. ODI, Oswestry Disability Index.

A total of four studies (20–23) evaluated the ODI (Figure 4A), which included 421 participants. The exercise therapy group demonstrated a significant improvement in quality of life according to the ODI (*p* < 0.00001). The meta-analysis applying the random-effects model showed that the ODI score of the treatment group was lower than that of the control group. This indicates that exercise therapy improves the quality of life of patients. There was a high degree of heterogeneity among the studies (I^2^ = 92%, *p* < 0.0001). Therefore, to validate the results, sensitivity analysis was performed (Figure 4B), which indicated that the results were robust despite the considerable heterogeneity among the studies.

#### 3.4.3 Joint ROM assessment

**Figure 5:**
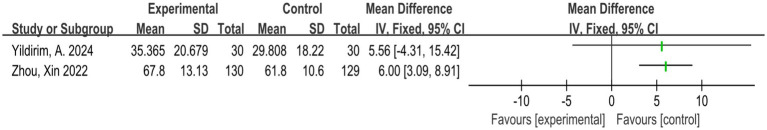
Direct pairwise random-effects meta-analysis of joint ROM. ROM, range of motion.

Two studies (21, 23) assessed the ROM of the lumbar spine in flexion, extension, and lateral flexion (right and left). As shown in Figure 5, 319 subjects participated. Because the sample sizes of the two studies were quite different, we did not combine the forest maps. The final results were: Yildirim et al. (21): SMD = 5.56 (95% CI: −4.31 to 15.42); Xin et al. (14): SMD = 6.00 (95% CI: 3.09 to 8.91).

#### 3.4.4 McGill pain questionnaire

**Figure 6:**
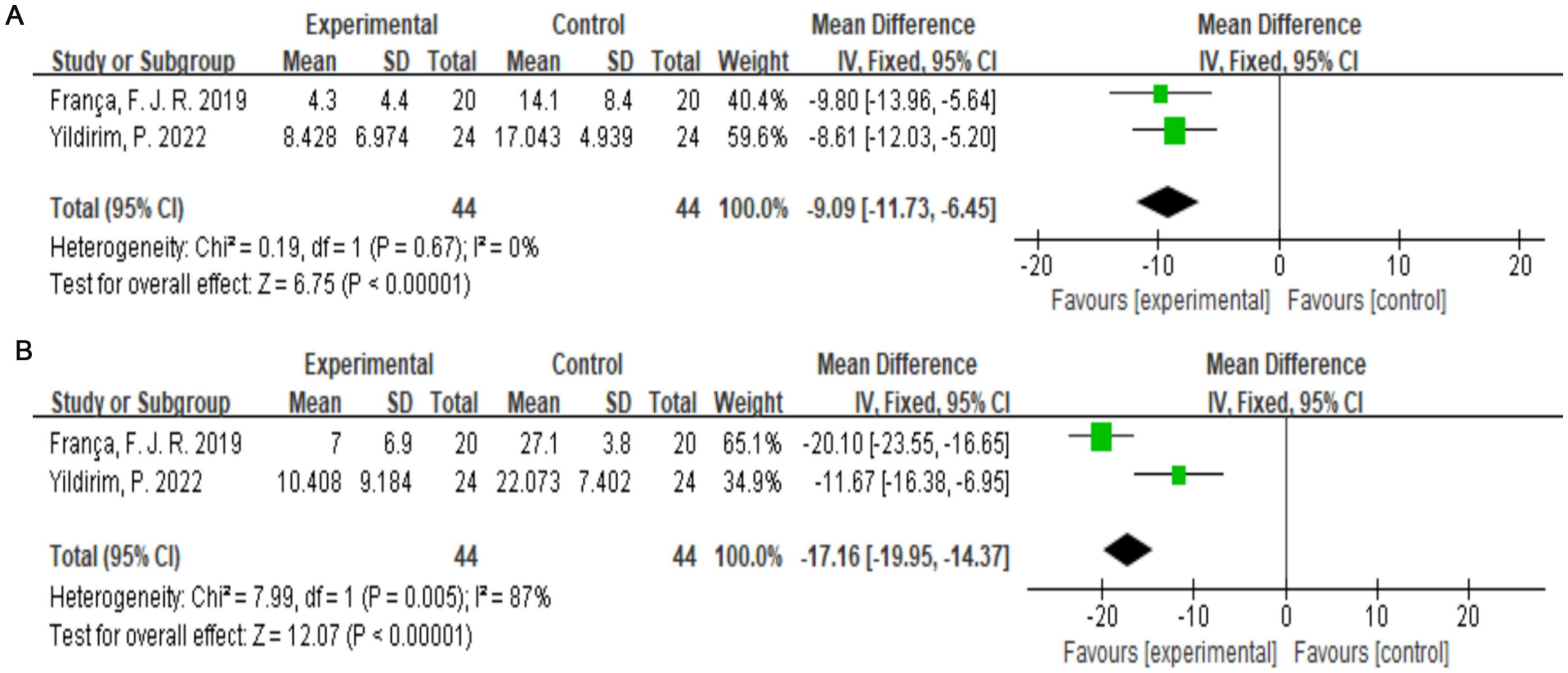
**(A)** Direct pairwise random-effects meta-analysis of McSensery. **(B)** Direct pairwise random-effects meta-analysis of McTotal.

The McGill Pain Questionnaire consists of 77 pain descriptions divided into four main categories (sensory, affective, evaluative, and miscellaneous) and 20 subcategories, each of which contains a minimum of two and a maximum of six words to which pain intensity values are assigned (25). Two studies (19, 22) with a total of 88 subjects were included. Combining the results of the two subanalyses (McSensery, McTotal) (Figures 6A,B), exercise therapy was effective in relieving pain in patients with LDH (*p* < 0.00001).

#### 3.4.5 SF-36

**Figure 7:**
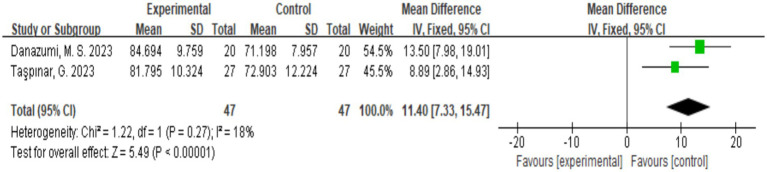
Direct pairwise random-effects meta-analysis of SF-36. SF-36, Short Form-36.

Two studies assessed the quality of life of patients according the SF-36 scale (18, 20), and a total of 94 participants were included. Figure 7 shows that the quality of life of the patients significantly improved after exercise therapy compared with the control group (*p* < 0.00001).

### 3.5 Safety assessment

None of the eight studies indicated any significant adverse reactions or symptoms of discomfort in participants treated with exercise therapy for LDH.

## 4 Discussion

### 4.1 Biological mechanisms underpinning the beneficial effects of exercise in the treatment of LDH

It is certain that exercise therapy has beneficial effects in patients with LDH. The normal intervertebral disc is mainly composed of the fibrous annulus, cartilaginous endplate, and nucleus pulposus. The fibrous annulus is a layered structure of ligaments that wraps around the nucleus pulposus and is mainly composed of type I collagen fibers. The cartilaginous endplate is composed of a small amount of hyaline cartilage located between the endplate of the vertebra and the nucleus pulposus. The nucleus pulposus is a hydrated structure composed mainly of proteoglycans scattered in an irregular network of type II collagen fibers (1).

The pathogenesis of LDH is complex and has not been fully clarified (26, 75). In recent years, a large number of experimental and clinical studies have shown that the pathogenesis, diagnosis, and treatment strategies for LDH have gradually improved. Chronic mechanical load accumulation and sudden overload injury of the lumbar intervertebral discs are the main causes of LDH (27). In general, there are two types of structural destruction of the intervertebral discs: endplate fracture and annulus fibrosus rupture (28). These fissures have been shown to exacerbate disc degeneration, but they do not seem to cause pain in the early stages (29). A previous study suggested that the pathogenesis of LDH could be explained by the theories of mechanical compression, inflammatory chemical stimulation, and autoimmunity (30). Exercise therapy to promote the recovery of LDH may be related to these three theories.

First, regarding the theory of mechanical compression, Wade et al. (31) believed that body position and load rate were key factors in the process of disc herniation. Experiments were conducted on the lumbar vertebrae of sheep with pre-existing defects in the central dorsal ring with different loading positions. Thirty sheep with lumbar vertebrae motion segments were subjected to varying loads in the dynamic disc loading simulator. The discs were scanned with high-resolution MRI before and after the test. It was found that the discs contained pre-existing characteristic defects in the central dorsal ring, suggesting that naturally occurring disc defects could serve as the starting site for herniation. Previous studies have found that mechanical nerve fiber deformation may also be the result of changes in the nerve root microcirculation, the occurrence of ischemia, and the formation of nerve edema, leading to impaired capillary blood flow and affecting the arterial blood supply (32, 33). Therefore, vertebral venous stasis may be an important cause of radicular pain. In this regard, long-term systematic exercise can reduce blood viscosity and improve blood circulation in a certain range, promoting local adaptation of the lesions (34). A previous meta-analysis and a clinical study concluded that exercise can reduce the pressure in the lumbar space, effectively improving the microcirculation at the site of the lesion obstruction (35, 36). This may be why exercise reduces neuromechanical compression.

The inflammatory state has been identified as a potential cause of pain in patients with LDH. Relevant animal experiments and clinical trials have supported the view that changes in inflammatory markers are related to the local inflammatory response in the blood and paravertebral muscles, and they may serve as analytical prediction targets for the presence of lower back pain (37–39). Dysregulated local inflammatory activity of the paravertebral muscles has been suggested as another possible predictor of lower back pain in patients with LDH. The results of a prospective cohort study (40) showed that in patients with LDH with high fat infiltration and reduced cross-sectional area, the expression of tumor necrosis factor (TNF) was elevated in those with lower back pain. In addition, TNF expression was closely related to lower back pain severity. Denatured adipose tissue is a major source of pro-inflammatory cytokines, which play an important role in various muscle pathologies. Data from animal models characterized by disc degeneration or lesions showed that elevated TNF expression in the adipose and connective tissues of the paravertebral muscles was significantly associated with the polarization of pro-inflammatory macrophages (37, 39). A related study observed higher TNF expression in the paravertebral muscles in individuals with high fat infiltration than in those with low fat infiltration (13). This strongly supports the conclusion that TNF promotes fat infiltration in the paravertebral muscles and reduces the multifidus muscle area. Other studies have confirmed that the upregulation of TNF expression is related to pain intensity (41–44). Moreover, Wang et al. (45) demonstrated that the serum levels of inflammatory factors (such as interleukin-6 and TNF-α) were negatively correlated with insulin-like growth factor (IGF)-1 levels. Therefore, IGF-1 may have an inhibitory effect on inflammatory factors such as TNF-α and interleukin-6. A previous study evaluating exercise interventions suggested that exercise may affect the IGF-1 level in the skeletal muscle and circulatory system (46). Singh et al. (47) found that IGF-1 expression was positively correlated with muscle strength through muscle biopsy and immunofluorescence experiments, indicating that exercise can significantly upregulate IGF-1 (48, 49). Intermittent aerobic exercise is more effective than continuous aerobic exercise (50). This may be one of the important biological mechanisms by which exercise improves inflammatory chemical stimulation.

A stray disc in the epidural space can cause an autoimmune response, leading to activation of the inflammatory response and formation of granulation tissue, which appears as a ring enhancement called the “bull’s eye sign” (1). Normal discs are avascular and have a unique structure that isolates the nucleus pulposus from the proprioceptive immune system. A normal stable disc suppresses the infiltration of immune cells and cytokines. The immune privilege of the intervertebral disc is attributed to the blood–nucleus pulposus barrier and local expression of Fas ligands (51–55). Fas ligands induce apoptosis of immune cells and vascular endothelial cells through complex signaling pathways, maintaining the immune privilege of the intervertebral disc and preventing intervertebral disc angiogenesis (56). When the herniated intervertebral disc is exposed to the immune microenvironment, this can trigger an autoimmune response and induce various physiological and pathological processes, such as neovascularization and immune cell infiltration. When the herniated disc tissue is extruded out of the epidural space, it induces an autoimmune response. The lymphocytes activate related factors secreted by macrophages, which promote the paracrine and autocrine activity of macrophages, thus accelerating their recruitment to the disc. This in turn induces pro-inflammatory factors, which affect angiogenesis, promote the expression of matrix metalloenzymes, and accelerate the apoptosis of the herniated disc nucleus pulposus cells. Interestingly, macrophages are key immunomodulators that trigger LDH reabsorption. An animal study (57) showed that nerve root compression in mice using degraded nucleus pulposus tissue triggered increased macrophage infiltration in the dorsal root ganglion and significant activation of the microglia in the dorsal horn of the spinal cord. By contrast, compression of nerve roots with undenatured nucleus pulposus tissue only resulted in transient sciatica with transient infiltration and activation of macrophages and microglia. The study also found that continued use of PLX5622, a specific colony-stimulating factor 1 receptor antagonist, reduced macrophages and microglia, thereby effectively alleviating LDH-induced sciatica. It was concluded that macrophages and microglia play important roles in the dorsal root ganglion after LDH.

Previous studies have suggested that there is a significant negative correlation between interleukin-1β and interleukin-17 concentrations and LDH status (58, 59). Moreover, the key immunosuppressive factor interleukin-10 is significantly positively correlated with LDH (60). Hoffman et al. (61) found that prolonged moderate-to-moderate exercise increased interleukin-10 secretion in mice. In other studies, regular exercise decreased interleukin-1β and interleukin-17, while increasing interleukin-10 (62–64). Some studies have shown that the influence of exercise on cytokines may be related to the intensity and type of exercise. For instance, Peake et al. (65) compared different running intensities and found that the expression of interleukin-10 was significantly increased in the high-intensity group, whereas there was no significant change in the other groups. Other studies have shown that interleukin-17 increased after high-intensity running, but it decreased after free exercise (66, 67). In summary, the changes in immunomodulators, such as interleukin-1β, interleukin-6, and interleukin-10, are closely related to the pathogenesis of LDH.

### 4.2 Application of traditional Chinese medicine exercises for the treatment of LDH

Traditional Chinese medicine exercises, including Tai Chi, Baduanjin, Yijinjing, and Wuqinxi, are characterized by deep breathing with the diaphragm, and slow, gentle, and symmetrical movements. These exercises emphasize breathing, vomiting, and mind control, which are conducive to musculoskeletal stretching, relaxation of the body and mind, and improvement in body function, and they have excellent effects in the treatment of chronic pain (68). A 2023 systematic review and meta-analysis (69) evaluated the effectiveness of traditional Chinese medicine exercises in reducing pain and disability in middle-aged and elderly patients with LDH. The study included 22 randomized controlled trials involving 1,931 patients. Four commonly used traditional Chinese exercises were evaluated, including Baduanjin, Yijinjing, Tai Chi, and Wuqinxi. Compared with the non-intervention group, the application of traditional Chinese medicine exercises had obvious advantages in improving the pain and other discomfort caused by LDH. Among them, Baduanjin and Yijinjing showed significant improvements in the VAS score and JOA score in patients with LDH. Tai Chi has also been shown to improve muscle strength and enhance lumbar stability. A retrospective cohort study by Deng et al. (70) found that daily practice of Tai Chi could delay the imaging changes observed in middle-aged and elderly people with LDH. Another study showed that Tai Chi significantly reduces acute muscle pain (71), and Baduanjin can significantly improve balance and leg muscle strength (72). Yijinjing improves skeletal muscle mass in middle-aged and elderly people with muscle loss (73) and relieves local tissue hardness (13). Wuqinxi can strengthen the lumbar muscles and increase spinal motion (74). All of these studies strongly support the potential advantages of traditional Chinese medicine exercise therapy for reducing pain and improving mobility.

### 4.3 Prospects

In this study, a subgroup-merging formula was introduced to more accurately reflect the outcome. Exercise therapy is an economical and effective adjuvant therapy that can effectively improve lumbar muscle strength and lumbar stability in patients with LDH. Future research should explore the relationship between macrophages and autoimmunity after exercise therapy in consideration of the fact that macrophages can trigger LDH reabsorption. Moreover, future research should further clarify which types of LDH are receptive to exercise therapy. Based on this direction, future trials could be conducted by designing a three-arm comparison (e.g., exercise vs. massage vs. exercise + massage) to clarify the synergistic mechanism and expand the research base to support the efficacy of exercise therapy and combination therapy.

### 4.4 Limitations

This study has some limitations. First, the number of randomized controlled trials included in this study was small, and thus the number of studies evaluating each of the assessment indicators was limited. Research limitations can significantly influence the interpretation and applicability of study findings, however, our study provides preliminary evidence to guide future studies, especially in understudied populations (such as those we excluded for severe disease, surgery, and older patients over 65 years of age). Limited by the research scale, the included studies were all aerobic exercise, so that there is certainly quite a bias to the research results, influenced by excluded exercise. Second, the accuracy of the data synthesis should be considered, and further high-quality randomized controlled trials are needed to improve the understanding of this topic in the future. Third, only three of the included studies described follow-up, and thus we lacked sufficient evidence of symptom improvement in patients with LDH treated with exercise therapy. Finally, because most of the included studies used combination interventions, the independent effects of exercise therapy need to be validated in our clinical cohort.

## 5 Conclusion

This study summarizes the evidence obtained from eight randomized controlled trials of exercise therapy for patients with LDH. This preliminary meta-analysis suggests that exercise therapy has the ability to improve pain and disability, and its efficacy was reflected using several assessment scales, including the VAS and the ODI. No adverse events were reported in the randomized controlled trials of exercise therapy for LDH, indicating the safety of this treatment approach. However, caution must be exercised when interpreting these findings owing to the limited number of studies and the high degree of heterogeneity among the studies with respect to some of the outcome metrics. In conclusion, the results of this study suggest that exercise therapy may be a viable and safe treatment option for patients with LDH.

According to the heterogeneity of exercise regiments, our findings primarily support the general benefits of exercise-based LDH interventions, so the clinician should tailor the type and intensity of exercise to the specific factors of the patient (such as pain tolerance, functional status). Exercise therapy has many advantages as a therapeutic intervention for LDH. It is simple, convenient, highly efficient, and universally available. The benefits of traditional Chinese medicine exercises should also be further explored as a potential therapy for the treatment of LDH.

## Data Availability

The original contributions presented in the study are included in the article/supplementary material, further inquiries can be directed to the corresponding authors.
